# Comparison of two different physical activity monitors

**DOI:** 10.1186/1471-2288-7-26

**Published:** 2007-06-25

**Authors:** David R Paul, Matthew Kramer, Alanna J Moshfegh, David J Baer, William V Rumpler

**Affiliations:** 1 Diet and Human Performance Laboratory, Beltsville Human Nutrition Research Center, Agricultural Research Service, United States Department of Agriculture, 308 Center Rd., Beltsville, MD, 20705, USA; 2 Department of Health, Physical Education, Recreation, and Dance, University of Idaho, P.O. Box 442401, Moscow, ID, 83844, USA; 3 Biometrical Consulting Service, Agricultural Research Service, United States Department of Agriculture, 10300 Baltimore Ave., Building 005, Beltsville, MD, 20705, USA; 4 Food Surveys Research Group, Beltsville Human Nutrition Research Center, Agricultural Research Service, United States Department of Agriculture, 10300 Baltimore Ave., Building 005, Beltsville, MD, 20705, USA

## Abstract

**Background:**

Understanding the relationships between physical activity (PA) and disease has become a major area of research interest. Activity monitors, devices that quantify free-living PA for prolonged periods of time (days or weeks), are increasingly being used to estimate PA. A range of different activity monitors brands are available for investigators to use, but little is known about how they respond to different levels of PA in the field, nor if data conversion between brands is possible.

**Methods:**

56 women and men were fitted with two different activity monitors, the Actigraph™ (Actigraph LLC; AGR) and the Actical™ (Mini-Mitter Co.; MM) for 15 days. Both activity monitors were fixed to an elasticized belt worn over the hip, with the anterior and posterior position of the activity monitors randomized. Differences between activity monitors and the validity of brand inter-conversion were measured by *t*-tests, Pearson correlations, Bland-Altman plots, and coefficients of variation (CV).

**Results:**

The AGR detected a significantly greater amount of daily PA (216.2 ± 106.2 vs. 188.0 ± 101.1 counts/min, P < 0.0001). The average difference between activity monitors expressed as a CV were 3.1 and 15.5% for log-transformed and raw data, respectively. When a conversion equation was applied to convert datasets from one brand to another, the differences were no longer significant, with CV's of 2.2 and 11.7%, log-transformed and raw data, respectively.

**Conclusion:**

Although activity monitors predict PA on the same scale (counts/min), the results between these two brands are not directly comparable. However, the data are comparable if a conversion equation is applied, with better results for log-transformed data.

## Background

Despite the fact that physical activity (PA) is considered an important factor in the reduction of the risk of developing many chronic diseases [1-3], scientists have struggled with the complexities associated with quantitatively measuring PA [4]. Due to the tendency of survey or self-report techniques to imprecisely estimate PA [4-7], the use of activity monitors (AM) as objective measures of PA has steadily increased since the 1980's [8]. Activity monitors, or accelerometers, are small devices worn by human subjects (generally on the hip or wrist) that continuously measure the acceleration of bodily movements. The data from AMs are sometimes reported as independent measures of PA, minutes spent in PA of different exercise intensities, and/or converted into estimates of energy expenditure [9-11].

The selection of one of the many different brands of AMs is dependant on cost, size, weight, performance characteristics, and validity/reliability [5,12,13]. Due to these differences, it is unlikely that any single brand of AM will be universally adopted, bringing into question whether the results of studies using different brands of AMs produce comparable results. The raw data from AM's are an arbitrarily scaled measure called "counts", for which no standard currently exists between the different manufacturers (the magnitude of the counts depend on the different electrical and/or mechanical characteristics of the AM). One study comparing different brands of AMs using a mechanical set up (not placed on human subjects) demonstrated that count data are not comparable [14]. However, this problem could be mitigated if data from one brand could be accurately converted to another, as long as the true relationship between PA and the counts from at least one AM brand was known.

In this investigation, we compared two of the most common AMs (Actigraph™ from Actigraph LLC and the Actical™ from the Mini-Mitter Co.) in 56 women and men that wore both AMs attached to the same belt over the course of 15 days. We wished to determine; (1) if the two brands produce different group mean predictions of PA (bias) and/or large within-subject differences (variance), and (2) if there are large biases and variance differences between the AMs, what is the result of converting the AM data from one brand to another?

## Methods

### Subjects

The subjects were 28 women and 28 men, aged 30 to 60 yrs, with a BMI of 26.3 ± 4.2 (women) and 27.9 ± 4.9 kg·m^-2 ^(men). The study protocol was approved by the Johns Hopkins University Bloomberg School of Public Health Committee on Human Research. Prior to participation, subjects provided written informed consent and received a medical evaluation by a physician that included measurement of blood pressure and analysis of fasting blood and urine samples to screen for presence of metabolic disease.

The AMs used in this comparison study were the Actigraph™ (AGR; Model 7164, Actigraph, LLC) and the Actical™ (MM; Mini-Mitter Co.). The AGR is a 51 × 41 × 15 mm uniaxial accelerometer that weights 43 g with a battery. The MM is a 28 × 27 × 10 mm omnidirectional accelerometer that weighs 17 g with a battery. More detailed information comparing the mechanical characteristics of the AMs is described elsewhere [12,14]. To collect comparative data, both AMs were placed on the same snuggly-fitting belt worn over the right hip, with the MM attached to the belt with Velcro and the AGR directly looped through the belt. To minimize the effects variations in AM placement [13,15], the position of each AM on the belt (anterior or posterior) was randomized. The AMs were set to read the data in 1-min intervals, with the internal timers of both AMs started at the exact same time. This procedure was carried out for 15 days, but the first and last days were discarded from the analysis because the subjects did not have the opportunity to wear the AMs for the entire 24 hr day (13 full days of data were used in the analysis). The AGR AMs were calibrated to the manufacturers specifications prior to being placed on the subjects; a calibration device for the MM was not available at the time of the study (however, these AMs were new from the manufacturer).

The minute-by-minute data were downloaded from each of the AMs individually, then the raw data were compiled into a master database using a custom computer program written in the SAS language [16] developed in our laboratory. To minimize the effect of zeroes produced due to AM removal, the only days accepted for analysis were those with a minimum of 14 hrs, assuming that sequences of 20 min of consecutive zeroes represented AM non-wear [17,18]. The mean hours of AM wear in the database was 16.9 ± 2.4 hrs/day. Data were analyzed as daily means, with the mean number of days of data per subject being 10.7 (total of 600 days of data). AMs were rotated through the subjects so that 18 of the AGRs and 28 of the MMs were worn by two different subjects.

### Statistical analyses

**Figure 1:**
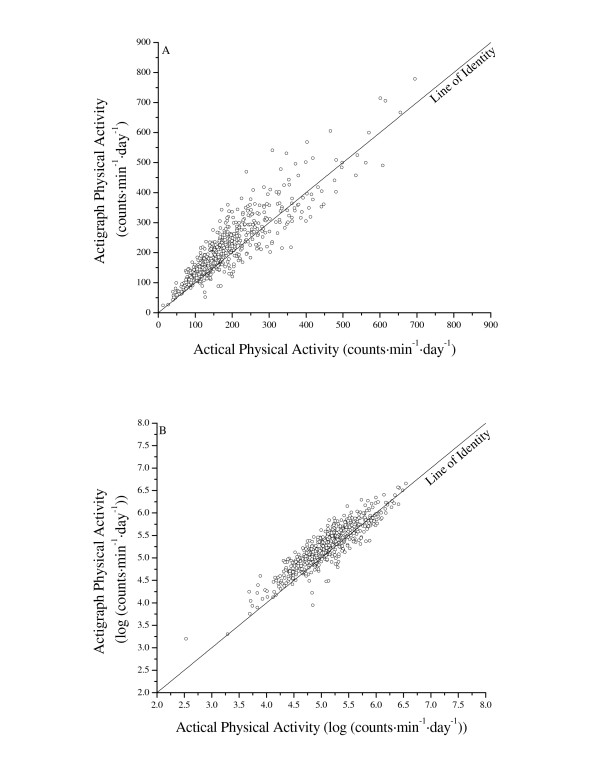
Relationships between physical activity predicted by Actigraph and Actical activity monitors for raw (A) and log-transformed (B) data (n = 56 subjects, 600 observation days).

Preliminary analyses detected significant heteroscedasticity (the daily count data were not normally distributed) [19] in the relationship between both AM brands (Figure 1A plots the results for the AM brands against each other), therefore the data were log-transformed prior to analysis (daily AM data were log-transformed as log (counts·min^-1^·day^-1^)). The log-transformation was effective at removing the heteroscedasticity (Figure 1B). We present results for both the raw and log-transformed data.

Mean values of PA for the two brands were compared using paired *t*-tests. The linear relationships between the brands of AMs were calculated using Pearson's correlation coefficient. Daily coefficients of variation (CV) were generated by dividing the standard deviation of (AGR - MM) by (AGR + MM)/2 times 100. The biases and variability between brands were also analyzed using Bland-Altman plots [20].

To inter-convert the data from the different AMs, one can develop correction factors based on the minute-by-minute data and/or the daily means. However, in order to generate a regression based on the minute-by-minute data, one must be certain that both AMs record PA at the same time. In other words, a "pulse" of PA from a subject must be detected in the same one minute interval by both AMs. Although both AMs were attached to the same belt (adjacent to each other) and the internal clocks were started at the same time, preliminary visual analyses indicated that pulses in PA were not being detected in the same one minute intervals as the collection period progressed. Thus, it appeared as though there was a "drift" in the time clocks of one or both of the AMs. To test the hypothesis that there was a drift in the internal clocks, a minute-by-minute CV was generated by dividing the standard deviation of (AGR - MM) by (AGR + MM)/2 times 100. These CV's were then averaged by day, and the differences between them were analyzed by a mixed model analysis of variance (Proc Mixed in SAS [16]). A Tukey's HSD test was used to detect post-hoc differences in the daily last squares means.

To develop a conversion equation for the daily means, slopes in the relationships between AM brands were measured in a mixed model analysis of variance (Proc Mixed). Other design effects such as AM position (which AM brand was placed anteriorly), AM ID (allowing individual AMs to have different intercepts), gender, and BMI were found not to be significant. Subject was modeled as a random effect. In turn, the slopes and intercepts estimated in the analysis were used to convert the data from one brand to the other. These estimates of PA were then compared using paired (by subject) *t*-tests. Analyses were performed on the daily raw count data, and when the daily data were log-transformed.

## Results

**Figure 2:**
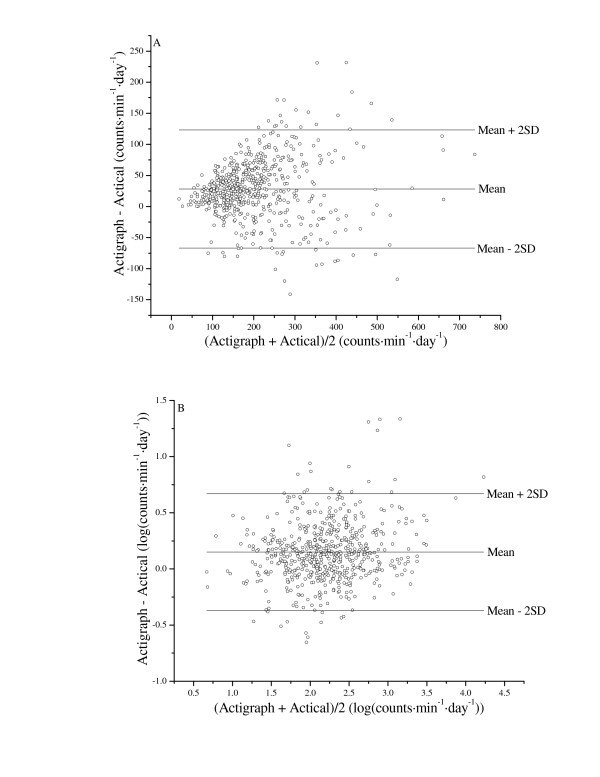
Bland-Altman comparisons between physical activity predicted by Actigraph and Actical activity monitors for raw (A) and log-transformed (B) data (n = 56 subjects, 600 observation days).

Daily PA estimates for the two different brands of AMs are presented in Table 1 and Figure 1. Despite the strong correlation between AMs, the AGR recorded significantly higher PA. The biases and variability is further demonstrated in Figure 2. However, both the bias and variability were reduced considerably when the daily count data were log-transformed prior to analysis.

**Table 1:** Comparisons between mean daily activity monitor data from the Actigraph and Actical, expressed as raw and log-transformed data (n = 56, total of 600 observation days).

	Actigraph counts·min^-1^·day^-1 ^mean (SD)	Actical counts·min^-1^·day^-1 ^mean (SD)	CV %	R
Raw	216.2 (106.2)	188.0 (101.1)*	15.5	0.90**
Log-transformed	5.26 (0.48)	5.11 (0.51)*	3.1	0.90**

*statistically significant, *t*-test (P < 0.0001)

**statistically significant, correlation (P < 0.0001)

Raw = raw activity monitor data.

Log-transformed = activity monitor data after log-transformation, log (counts·min^-1^·day^-1^).

CV = coefficient of variation of the difference between Actigraph and Actical.

R = Pearson Product Moment Correlation Coefficient.

**Figure 3:**
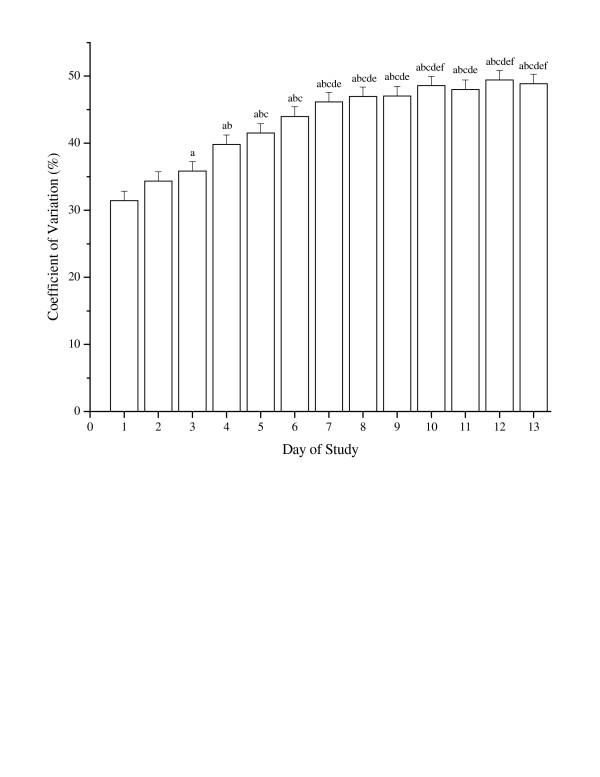
Minute-by-minute activity monitor comparisons (averaged by day) between Actigraph and Actical activity monitors (expressed as a coefficient of variation). ^a ^significantly different when compared to Day 1, p < 0.05. ^b ^significantly different when compared to Day 2, p < 0.05. ^c^significantly different when compared to Day 3, p < 0.05. ^d^significantly different when compared to Day 4, p < 0.05. ^e^significantly different when compared to Day 5, p < 0.05. ^f^significantly different when compared to Day 6, p < 0.05. Coefficient of Variation (%) = minute-by-minute standard deviation of (AGR - MM) divided by (AGR + MM)/2 times 100.

The minute-by-minute CV's (averaged by day) are shown in Figure 3. This analysis demonstrates that the differences in the minute-by-minute data between the AM brands became more pronounced as the collection period progressed, showing the time discrepancy between the AM brands. Therefore, it did not make sense to generate prediction equations to convert minute-by-minute data between the AM brands.

Linear regressions representing the relationship between the AM brands for daily data produced the following equations:

Actigraph (counts·min^-1^·day^-1^) = 38.5168 + (0.9467 * Actical (counts·min^-1^·day^-1^))

log (Actigraph (counts·min^-1^·day^-1^)) = 0.9401 + (0.8470 * log (Actical (counts·min^-1^·day^-1^)))

Actical (counts·min^-1^·day^-1^) = -2.9883 + (0.8789 * Actigraph (counts·min^-1^·day^-1^))

log (Actical (counts·min^-1^·day^-1^)) = -0.0616 + (0.9808 *log (Actigraph (counts·min^-1^·day^-1^)))

Table 2 demonstrates the effect of using the above regression equations to convert the daily PA data from one brand to the other. When using the raw data, the between brand bias is removed (by the intercept term), but CV's are still large (11–12%). However, performing the conversions with log-transformed data greatly reduces the variability (CV's less than 2.5%). There was a significant subject effect (Wald test, p < 0.001), indicating that the intercepts of the conversion equations differed by subject. Theoretically, if one knew which subject wore the AM and their subject specific intercept value, one could do better than by using the average intercept value. In the typical research setting where one does not know subject specific intercepts, however, the average intercept value (given in the equations above) would be used.

**Table 2:** Comparisons between mean daily activity monitor data from the Actigraph and Actical, with- and without conversion by a regression equation, expressed as raw and log-transformed data (n = 56, total of 600 observation days).

	Actigraph			Actical		
	Measured	Predicted	CV	Measured	Predicted	CV

	counts·min^-1^·day^-1^		%	counts·min^-1^·day^-1^		%

Raw	216.2	216.5	11.7	188.0	187.0	12.2
Log-transformed	5.27	5.26	2.2	5.11	5.10	2.4

Raw = raw activity monitor data.

Log-transformed = activity monitor data after log-transformation.

Measured = measured PA data.

Predicted = prediction of PA based on the conversion of one brand to the other using a conversion equation.

CV = coefficient of variation of the difference between measured PA ("Measured ") and that predicted ("Predicted") by conversion of one brand to the other.

## Discussion

The principal finding of this investigation was that the output from the AGR and MM, both purportedly measuring PA, differed significantly. Although the raw data outputs from both AMs are "counts", these units of PA can differ between manufacturers, due to the A/D conversion, sensors, and amplification factors [12]. Therefore, it is not surprising that a conversion equation was needed to make the brand means equivalent. Fortunately, the conversion of the daily PA data between AM brands (averaged over subjects), using the proposed conversion equations, produces estimates that are free of bias and with low variability (provided that the daily data are log-transformed prior to conversion). This means that converting between brands with the conversion equations will not only produce accurate group mean estimates of PA, but individual-subject data also can be converted successfully (CV of 2 – 3%).

Although it would be of interest to be able to convert the minute-by-minute data from one brand to another, the results of our analyses indicate this was not possible. The development of this type of a regression equation is dependent on a very close matching of the internal clocks between the AM brands. Figure 3 indicates this was not the case, and there was drift in the minute-by-minute time periods as the study progressed. Adapting our regression equations for minute-by-minute data is possible, but only if the internal clocks of both AM brands are accurate. Similarly, minute-by-minute regression equations from a mechanical set up carried over a short period of time (such as Esliger and Tremblay [14]) could be generated, but the accuracy of the conversions would be reduced over time unless the internal clocks were kept calibrated to each other. Drift is less of a problem when summing over long periods of time, since it is only pulses of activity near the end of the time period that may be incorrectly attributed to one of the two sequential time periods.

It is clear from Tables 1 and 2, and Figure 1 that these data are not normally distributed, and that a log-transformation will help satisfy the normality of residuals assumption of regression. Clearly the conversion bias is much less when working with log-transformed data. However, since a bias is introduced when back-transforming to the original scale, many investigators prefer to work with data on the original scale. We investigated a back-transforming procedure that includes a bias correction using the residual variance [21], but since the residual variances were small, there was only a negligible improvement on the back-transformed estimates of the daily means (CVs were still 11–12%). Therefore, we suggest using the data on the log-transformed scale when performing analyses.

## Conclusion

This study confirms the findings of Esliger and Tremblay [14], who demonstrated that the PA data produced by the AGR and MM (using a mechanical setup, not placed on human subjects) are not comparable. However, we show that the daily AM counts from one brand of AM can readily be converted to another. Although the inter-conversion between AM brands appears to be reasonable, the approach must be carefully applied. First, we ensured that the subjects wore the AMs on a regular basis. We estimated that the subjects in this study wore the AM's approximately 17 hrs/day (all days with less than 14 hrs/day were discarded). If the conversion is applied to daily data where the adherence is much less than 17 hrs/day, it is likely that there will be more error when converting from one brand to another or estimating PA [18].

Given the feasibility of this approach, we hope that additional comparisons between other AM brands will be performed under similar free-living conditions in order better measure PA and thus better understand the relationships between PA and chronic disease.

## Abbreviations

PA Physical activity

AM Activity monitor

AGR Actigraph activity monitor

MM Actical activity monitor

CV coefficient of variation

SD standard deviation

## Competing interests

The author(s) declare that they have no competing interests.

## Authors' contributions

DRP was responsible for data collection, statistical analysis and manuscript preparation. MK was responsible for statistical analysis and manuscript preparation. AJM, DJB, and WVR were responsible for the study design and manuscript preparation. All authors have read and approved the final manuscript.

## Pre-publication history

The pre-publication history for this paper can be accessed here:


